# Intratumoral depletion of regulatory T cells using CD25-targeted photodynamic therapy in a mouse melanoma model induces antitumoral immune responses

**DOI:** 10.18632/oncotarget.17663

**Published:** 2017-05-07

**Authors:** Dong Sun Oh, Heegon Kim, Ji Eun Oh, Hi Eun Jung, Yun Soo Lee, Ji-Ho Park, Heung Kyu Lee

**Affiliations:** ^1^ Biomedical Science and Engineering Interdisciplinary Program, Korea Advanced Institute of Science and Technology, KAIST, Daejeon, 34141, Republic of Korea; ^2^ Graduate School of Medical Science and Engineering, KAIST, Daejeon, 34141, Republic of Korea; ^3^ Department of Bio and Brain Engineering, KAIST, Daejeon, 34141, Republic of Korea; ^4^ KAIST Institute for Health Science and Technology, KAIST, Daejeon, 34141, Republic of Korea

**Keywords:** photodynamic therapy, immunotherapy, Treg, CD25, Chlorin e6

## Abstract

Tumor immunotherapy aims to overcome the immunosuppressive microenvironment within tumors, and various approaches have been developed. Tumor-associated T regulatory cells (Tregs) suppress the activation and expansion of tumor antigen-specific effector T cells, thus, providing a permissive environment for tumor growth. Therefore, optimal strategies need to be established to deplete tumor-infiltrated Tregs because systemic depletion of Tregs can result in reduced anti-tumor effector cells and autoimmunity. Here, to selectively deplete Tregs in tumors, we intratumorally injected anti-CD25 antibodies conjugated to Chlorin e6 (Ce6), a photosensitizer that absorbs light to generate reactive oxygen species. Local depletion of tumor-associated Tregs with photodynamic therapy (PDT) inhibited tumor growth, which was likely due to the altered tumor immune microenvironment that was characterized by increased infiltration of CD8+ effector T cells and the expression of IFN-γ and CD107a, which is a cytolytic granule exocytosis marker in tumor tissues. Furthermore, PDT-induced intratumoral Treg depletion did not influence adaptive immune responses in a murine influenza infection model. Thus, our results show that intratumoral Treg-targeted PDT could specifically modulate tumor microenvironments by depleting Tregs and could be used as a novel cancer immunotherapy technique.

## INTRODUCTION

Tumor immunotherapy using antibodies, tumor vaccines, and cell-based therapies has been developed to eliminate tumors and to suppress tumor metastasis [1–3]. These tumor immunotherapies result in fewer side effects and are less invasive than conventional tumor therapies [4]. Recent advances in immunotherapies targeting immune-checkpoint molecules or immunosuppressive cells, such as regulatory T cells (Tregs) or myeloid-derived suppressor cells, have been highlighted in tumor treatments [5]. Tregs are considered one of the most significant targets as they play a major role in maintaining immunosuppressive microenvironments within tumors [6, 7].

Though many advances have been made, there are several issues that must be overcome to achieve successful tumor immunotherapies [8–10]. Most importantly, systemic depletion of Tregs has the potential to induce severe autoimmune diseases and hyper-immune responses against other pathogenic infections. Therefore, successful immunotherapy requires specific local and selective depletion of Tregs to avoid these side effects and to protect homeostasis in other organs [11].

Photodynamic therapy (PDT) exploits non-toxic photosensitizers that absorb light to generate reactive oxygen species (ROS) that can kill tumor cells by inducing apoptosis or necrosis [12, 13]. Light-dependent activation of photosensitizers enables site-specific treatment of tumors and helps reduce systemic toxicity compared to conventional chemotherapies [14, 15]. In addition, conjugation of photosensitizers to targeting agents, such as antibodies, could provide cell-specificity and/or site-specificity, further reducing off-target effects [16, 17]. Similarly, antibodies targeting immune cells could be exploited for selective depletion of target cells in tumor microenvironments to investigate their functions and therapeutic effects [18].

Here, we intratumorally injected Chlorin e6 (Ce6) conjugated to an anti-CD25 monoclonal antibody (anti-CD25-Ce6) to locally deplete CD4^+^Foxp3^+^ Tregs. This anti-CD25-Ce6-targeted PDT technique induced apoptosis and depletion of Tregs in tumors and inhibited tumor growth in a murine melanoma model. These effects are attributed to the altered immune microenvironment of the tumors, which was characterized by increased infiltration of CD8^+^ effector T cells and increased IFN-γ and CD107a production in tumor tissues. Furthermore, local depletion of Tregs via PDT did not influence adaptive immune responses against flu infection, suggesting that anti-CD25-Ce6-targeted PDT induced anti-tumoral immunity without altering systemic immune responses.

## RESULTS

### Anti-CD25-Ce6-targeted PDT induces apoptosis in CD4^+^Foxp3^+^ cells *in vitro*

**Figure 1:**
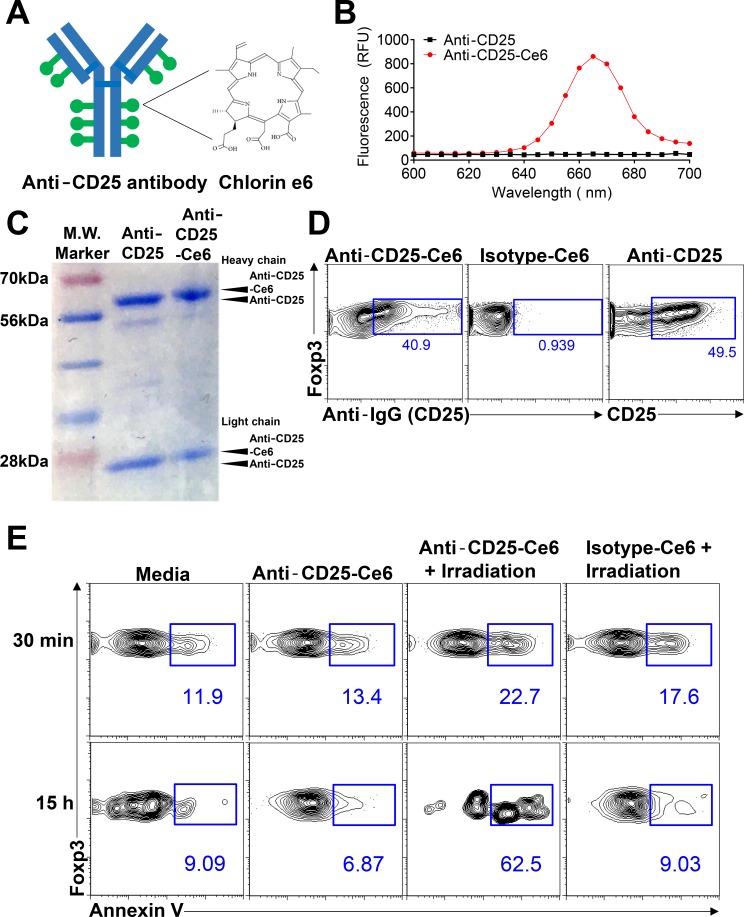
Anti-CD25-Ce6-targeted PDT induces apoptosis of target cells *in vitro* (**A**) Schematic of anti-CD25-Ce6 synthesization for treatment of mouse melanoma. Anti-CD25 antibody that can target Tregs are conjugated with Ce6 dye. (**B**) Fluorescence spectra of anti-CD25-Ce6 and anti-CD25 solutions with 400-nm excitation. (**C**) Anti-CD25-Ce6 and anti-CD25 was run on SDS-PAGE and stained with Coomassie blue dye. (**D**) CD4^+^Foxp3^+^ cells were stained with anti-CD25-Ce6 or isotype-Ce6 conjugate, followed by APC-conjugated goat anti rat secondary antibody. The binding efficiency of each conjugate was determined by flow cytometry. (**E**) CD4^+^Foxp3^+^ cells were irradiated with a 660-nm laser. Cell apoptosis was observed at 30 min or 15 h after irradiation by detecting annexin V expression using flow cytometry. Data are representative of three or four independent experiments.

Our first aim was to eliminate intratumoral Tregs by employing anti-CD25 antibody conjugated to Ce6 (anti-CD25-Ce6; Figure 1A). CD25 is a significant marker for Tregs and has, thus, been widely used for depletion of Tregs [19–22]. Ce6 is a photosensitizer that can generate ROS or heat when excited under irradiation by a 660-nm laser; the ROS generated can induce apoptosis in cells [23, 24]. For the conjugation of Ce6 to anti-mouse CD25 antibody, the carboxylic groups of Ce6 were linked to the antibody's amine groups using N-hydroxysuccinimide and 1-ethyl-3-(3-dimethylaminopropyl) carbodiimide. The number of Ce6 molecules conjugated to each anti-CD25 antibody molecule was estimated by analyzing the absorbance data. Approximately 10 Ce6 molecules were conjugated to each antibody molecule (Figure 1B). Protein gel electrophoresis showed that the molecular weight of anti-CD25-Ce6 was slightly increased compared with anti-CD25 antibody in terms of Ce6 attachment to the heavy and light chains of the CD25 antibody (Figure 1C).

To confirm that anti-CD25-Ce6 binds Tregs effectively, CD4^+^ T cells were sorted from spleen of Foxp3-GFP mice and stained with anti-CD25-Ce6, Ce6-conjugated isotype-control antibody (isotype-Ce6), or dye-conjugated anti-CD25 monoclonal antibody (anti-CD25). Binding of each conjugate was detected with a fluorochrome-conjugated anti-rat IgG antibody. The anti-CD25-Ce6 conjugate bound CD4^+^Foxp3^+^ cells at a comparable rate to anti-CD25, while the isotype-Ce6 did not (Figure 1D).

We next examined the selective phototoxicity of anti-CD25-Ce6 against CD4^+^Foxp3^+^ cells. CD4^+^ T cells were sorted from spleens of Foxp3-GFP mice and treated with anti-CD25-Ce6 or isotype-Ce6. For PDT, a 660-nm laser was used to irradiate each well for 5 min. Apoptosis was measured at 30 min and 15 h after irradiation by detecting annexin V expression using flow cytometry. At both time points, anti-CD25-Ce6 induced apoptosis in CD4^+^Foxp3^+^ cells more effectively than did isotype-Ce6 (Figure 1E). Collectively, these results suggest that anti-CD25-Ce6 can selectively bind CD4^+^Foxp3^+^ cells in tumors and induce apoptosis under irradiation.

### Anti-CD25-Ce6-targeted PDT induces CD4^+^Foxp3^+^ Treg depletion and produces tumor regression *in vivo*

**Figure 2:**
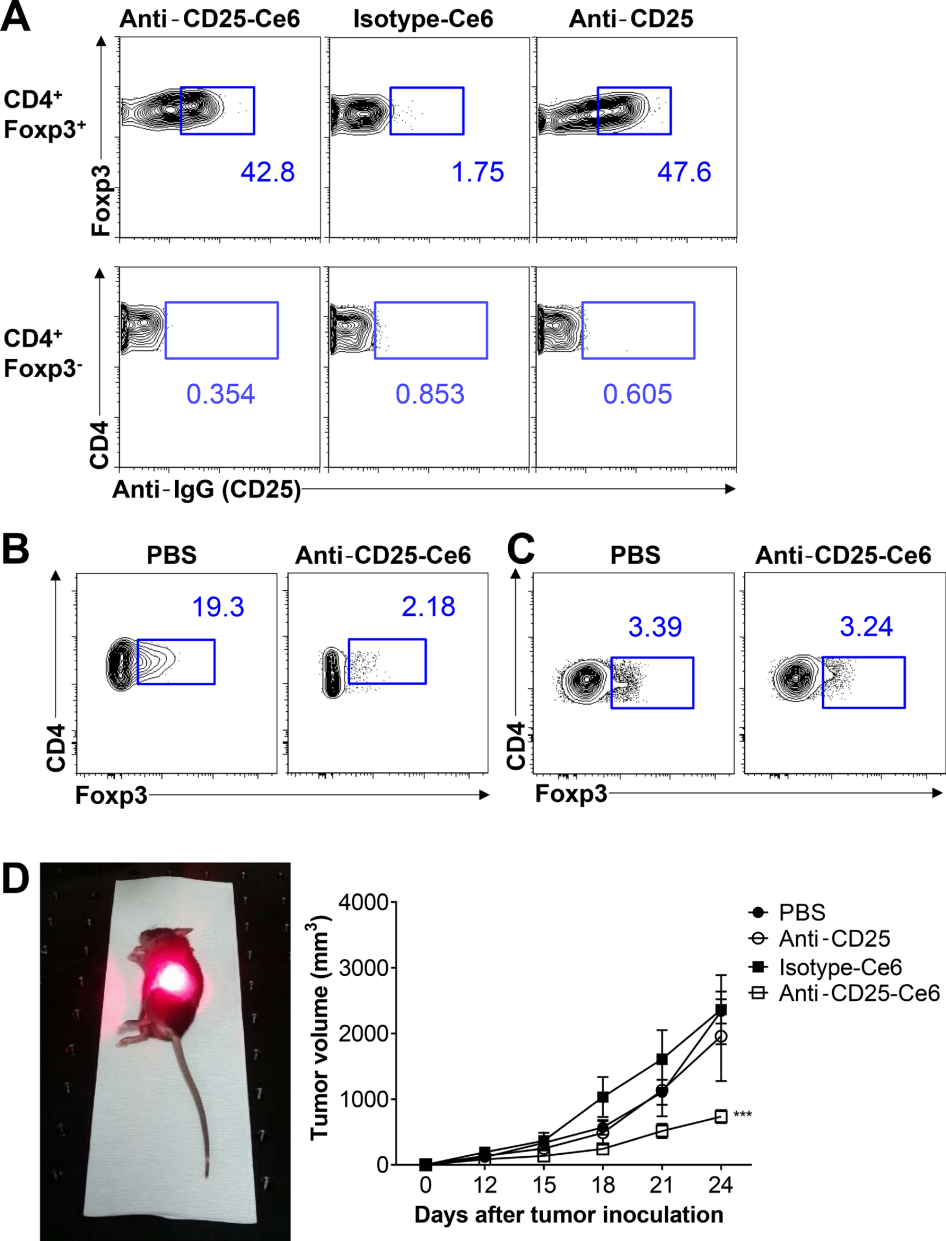
Anti-CD25-Ce6-targeted PDT induces depletion of intratumoral Tregs and regression of B16-F10 melanoma *in vivo* (**A**) Anti-CD25-Ce6 or isotype-Ce6 conjugate was injected into B16-F10 tumor-bearing mice. After 30 min, the binding efficiency of each complex was determined by flow cytometry following staining of fluorochrome-conjugated goat anti-rat IgG isotype-specific secondary antibody. (**B** and **C**) Ten days after B16-F10 melanoma cell transplantation, anti-CD25-Ce6 or PBS was injected intratumorally and tumors were irradiated with a 660-nm laser for 20 min. Tumors were irradiated twice at a 2-day interval. B. Intratumoral or C. draining lymph node CD4^+^Foxp3^+^ Tregs were monitored using flow cytometry. (**D**) PBS, anti-CD25 antibody, isotype-Ce6, or anti-CD25-Ce6 was injected intratumorally and PDT was conducted four times at 2-day intervals. Tumor growth was measured using a digital caliper. (*n* = 5–6 mice per group; two-way ANOVA, ***P < 0.001; error bars represent SEM). Data are representative of two or three independent experiments.

Intravenous injection of anti-CD25 antibody for systemic depletion of Tregs results in autoimmune diseases [11]. To overcome this harmful side effect, we performed intratumoral injection of anti-CD25 antibody to locally and selectively deplete Tregs within the tumor microenvironment. To confirm binding of anti-CD25-Ce6 to Tregs *in vivo*, anti-CD25-Ce6 was injected intratumorally in mice bearing B16-F10 melanoma. Tumors were collected 30 min after injection. Analysis of antibody binding revealed that intratumoral Tregs were effectively targeted by anti-CD25-Ce6 (Figure 2A).

To determine if tumor irradiation following injection of anti-CD25-Ce6 into tumors can selectively deplete intratumoral Tregs, we collected tumors following PDT (irradiation twice at a 2-day interval). Intratumoral Tregs were effectively reduced after irradiation (Figure 2B), while Tregs in the tumor-draining lymph node showed no significant changes (Figure 2C).

PDT effectively depleted tumor infiltrated CD4^+^ CD25^+^ Foxp3^+^ Treg, as well as CD4^+^ CD25^+^ Foxp3^-^ T cells that exhibit pathologic features and have a potential to become Tregs [25, 26] (Supplementary Figure 1). Overall, our results show that local and selective depletion of CD4^+^ CD25^+^ Foxp3^+^ Tregs was achieved *in vivo*.

Next, we investigated whether intratumoral depletion of Tregs can inhibit tumor growth. We performed PDT on mice bearing B16-F10 melanoma (four times total at 2-day intervals). Measurement of tumor volumes revealed that the anti-CD25-Ce6-injected mice showed significant inhibition of tumor growth, while isotype-Ce6 or free anti-CD25 showed no therapeutic effect (Figure 2D). Collectively, anti-CD25-Ce6 effectively bound and killed intratumoral Tregs *in vivo* and effectively inhibited tumor growth.

### Anti-CD25-Ce6-targeted PDT induces CD8^+^ T-cell tumor infiltration

**Figure 3:**
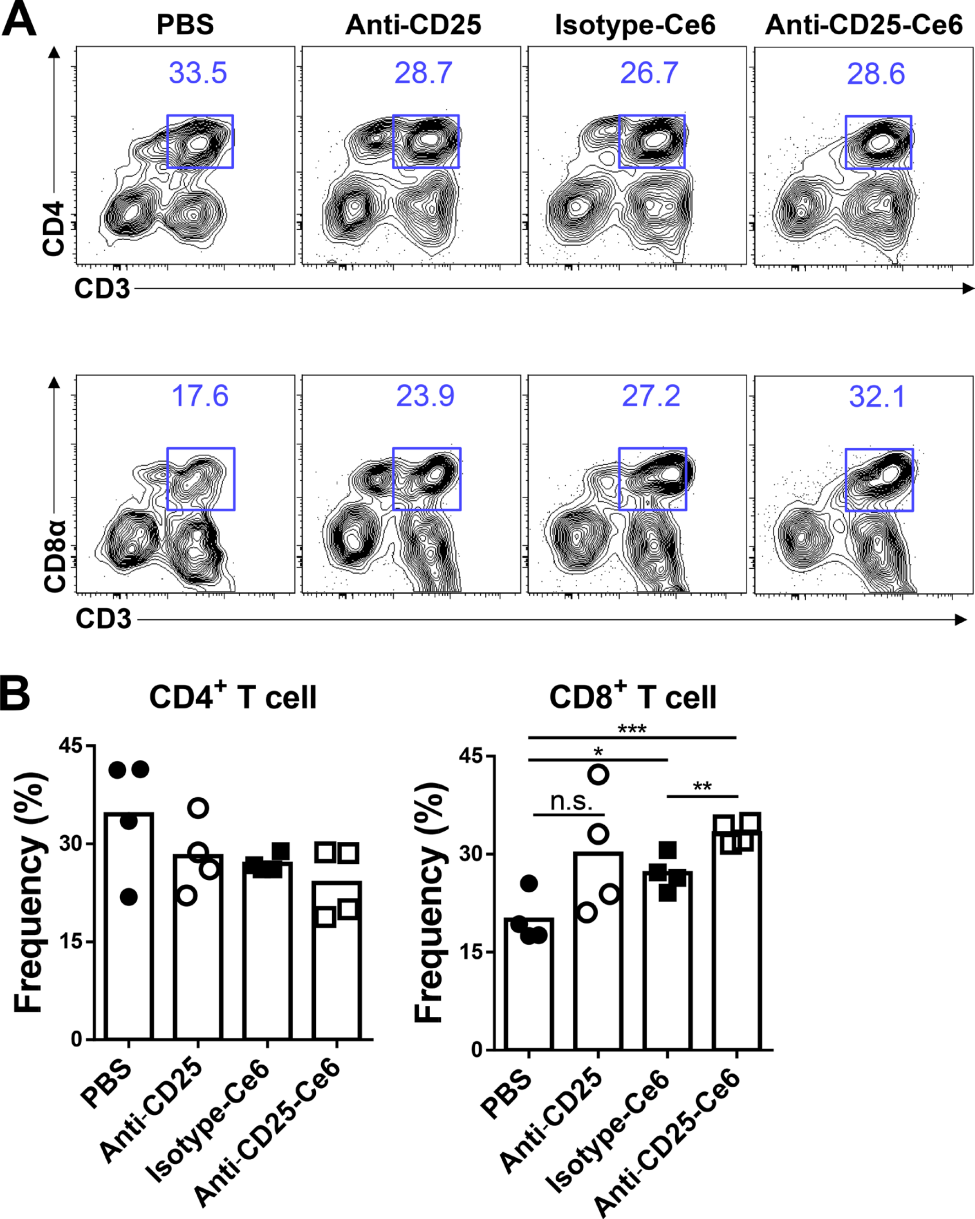
Anti-CD25-Ce6-targeted PDT induces CD8^+^ T-cell tumor infiltration (**A** and **B**) Ten days after tumor inoculation, PBS, anti-CD25 antibody, isotype-Ce6, or anti-CD25-Ce6 was injected intratumorally, after which PDT was performed twice at a 2-day interval. (A) Tumor-infiltrated T cells were examined using flow cytometry. (B) Results shown as bar graphs. (*n* = 4 mice per group; Student's *t* test, *P < 0.05, **P < 0.01, ***P < 0.001; error bars represent SEM). Data are representative of three independent experiments.

In anti-tumor immune responses, CD8^+^ cytotoxic T cells are a key eradicator of tumor cells. Several studies have shown that Treg depletion induces activation of cytotoxic CD8^+^ T cells and enhances infiltration of these cells into tumors [27, 28]. To determine if CD8^+^ cytotoxic T cells also infiltrate tumors after anti-CD25-Ce6-targeted PDT, we subcutaneously inoculated mice with B16-F10 melanoma cells. Ten days after tumor inoculation, PBS, isotype-Ce6, anti-CD25, and anti-CD25-Ce6 complex were injected intratumorally and tumors were irradiated with a 660-nm laser for 20 min. PDT was conducted twice at a two-day interval. Tumor-infiltrated CD4^+^ T cells and CD8^+^ T cells were monitored using flow cytometry. Tumor-infiltrated CD4^+^ T-cell levels were not significantly different between treatment groups. However, CD8^+^ T-cell infiltration was elevated more in anti-CD25-Ce6-treated mice than in control (PBS, isotype-Ce6, and anti-CD25-treated) mice (Figure 3A and 3B). Thus, these results show that effective depletion of intratumoral Tregs through anti-CD25-Ce6-targeted PDT enhances anti-tumor immunity by inducing CD8^+^ T-cell infiltration.

### Anti-CD25-Ce6-targeted PDT induces cytotoxic T-cell responses and polyfunctionality

**Figure 4:**
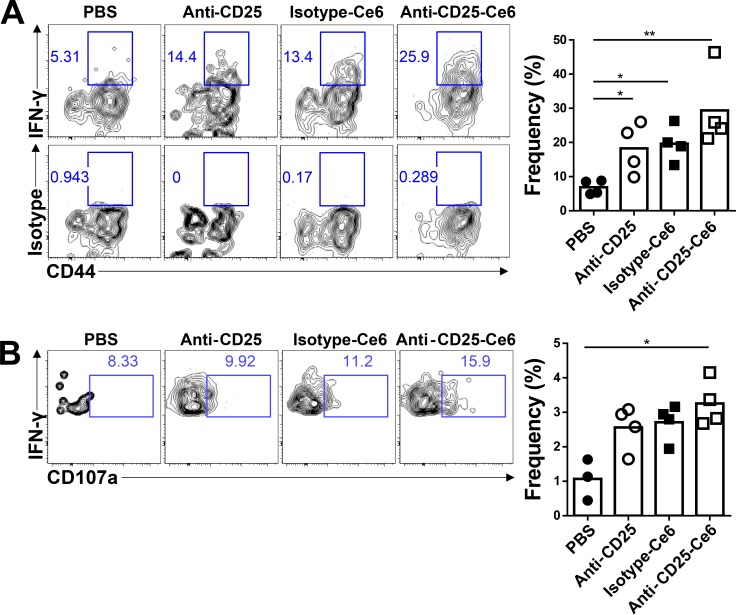
Anti-CD25-Ce6-targeted PDT induces cytotoxic T-cell responses and T-cell polyfunctionality (**A** and **B**) Ten days after B16-F10-cell inoculation, PBS, anti-CD25 antibody, isotype-Ce6, or anti-CD25-Ce6 was injected intratumorally and PDT was performed twice at a 2-day interval. (A) IFN-γ^+^- or (B) IFN-γ^+^CD107a^+^-expressing activated tumor-infiltrated CD8^+^ T-cell populations were assessed by intracellular cytokine staining following stimulation with phorbol myristate acetate/ionomycin. (*n* = 4 mice per group; Student's *t* test, *P < 0.05, **P < 0.01). Data are representative of three independent experiments.

Recent studies have demonstrated that tumor-infiltrated CD8^+^ T cells display several functional impairments, especially in their polyfunctional cytokine production that includes IFN-γ, TNF-α, and CD107a, which are high-quality effectors [29]. Tregs contribute to the suppressed polyfunctionality of cytotoxic CD8^+^ T cells [7]. Based on the hypothesis that local depletion of Tregs could recover the polyfunctionality of CD8^+^ T cells, we examined the functionality of tumor-infiltrated CD8^+^ T cells by measuring cytokine production. Ten days after tumor inoculation, anti-CD25-Ce6 was injected intratumorally and PDT was conducted twice at a 2-day interval. The anti-CD25-Ce6-treated mice showed the most significant increase in IFN-γ production compared with anti-CD25- and isotype-Ce6-treated mice (Figure 4A). Similarly, the IFN-γ^+^CD107a^+^CD8^+^ polyfunctional cytotoxic T-cell population was significantly increased in the anti-CD25-Ce6-treated mice (Figure 4B). Thus, Treg depletion through anti-CD25-Ce6-targeted PDT increased IFN-γ production by CD8^+^ T cells and enhanced their polyfunctionality.

### Anti-CD25-Ce6-targeted PDT does not affect the adaptive immune response against influenza infection

Elimination of Tregs through systemic administration of monoclonal antibodies may reduce tumor masses by inducing anti-tumor immunity [22]. However, systemic Treg depletion results in severe side effects, such as autoimmune responses or hyper-immune responses against other pathogen infections [30, 31]. Therefore, these side effects are a major obstacle for clinical application of systemic Treg-targeting drugs. Our strategy that uses antibody-targeted PDT to locally and selectively deplete Tregs has the advantage of selectively targeting tumor-infiltrated Tregs, the most significant suppressor of anti-tumor immune responses in tumor microenvironments.

**Figure 5:**
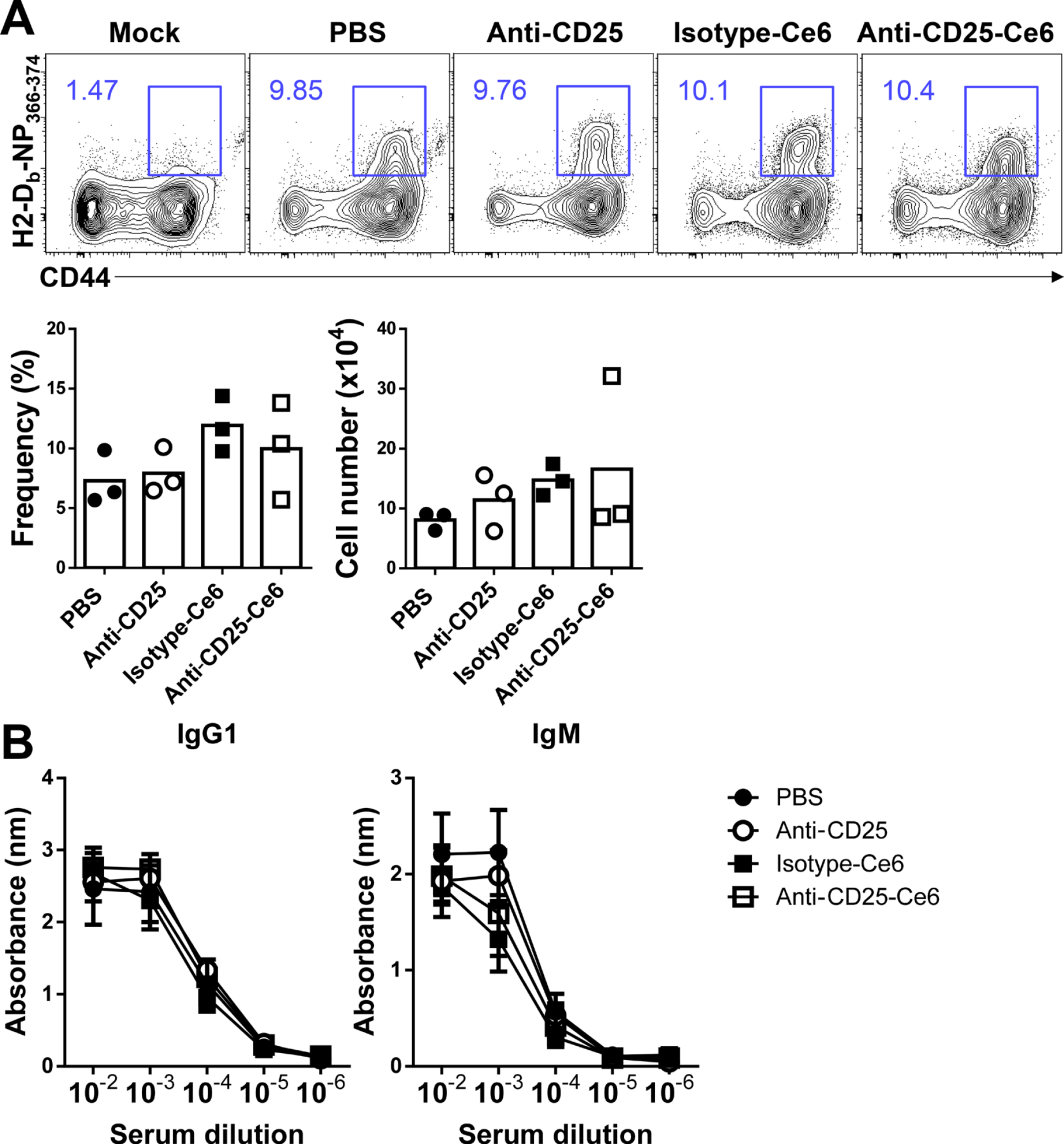
Anti-CD25-Ce6-targeted PDT does not affect the adaptive immune response against influenza infection (**A**) Mice were intranasally administered PR8 5 days after B16-F10 inoculation. Six days after PR8 infection, PDT was performed twice at a 2-day interval. PR8 NP_366-374_-specific CD8^+^ T cells in lungs were monitored using flow cytometry with the H2-Db-NP_366-374_ pentamer. (**B**) Bar graph of the frequency of PR8 NP_366-374_-specific CD8^+^ T cells. (*n* = 3 mice per group; Student's *t* test; error bars represent SEM). Data are representative of two independent experiments.

To verify that our therapy did not alter systemic immune responses, we utilized a mouse influenza infection model to determine if the influenza-specific immune response was altered following anti-CD25-Ce6-targeted PDT. Mice transplanted with B16-F10 melanoma were intranasally infected with PR8 virus. After two PDT treatments, PR8 NP3_66–374_-specific CD8^+^ T cells in the lungs of each mouse were monitored by flow cytometry using H2-Db-NP_366–374_ pentamers. The frequency and absolute cell number of influenza antigen-specific CD8^+^ T cells in the lungs of anti-CD25-Ce6-targeted PDT-treated mice were not significantly affected compared with the control groups (Figure 5A). In the case of intranasal PR8 infection after PDT treatment, there were no changes in the frequency or absolute cell number of influenza antigen-specific CD8^+^ T cells in the lungs (Supplementary Figure 2). Additionally, the humoral immune response against PR8 was also normal (Figure 5B). Together, these results suggest that our anti-CD25-Ce6-targeted PDT only modulated the tumor microenvironment through local depletion of Tregs, that resulted in tumor growth inhibition without affecting the adaptive immune response to influenza infection.

## DISCUSSION

Many attempts to treat solid tumors by therapeutic vaccination have failed due to a poor understanding of tumor-induced immuno-suppressive microenvironments [32]. Tregs are essential for the regulation of autoimmune responses and induce tumor immune tolerance [33]. Therefore, Tregs are a promising target in the tumor microenvironment and the depletion of Tregs induces immune responses against tumors [34]. However, there are limitations to the current Treg depletion techniques in that systemic depletion of Tregs has the potential to induce severe autoimmune inflammation and hyper-immune responses against pathogen infections. Thus, an approach that locally and selectively depletes tumor-associated Tregs to avoid these severe side effects and protects the homeostasis of other organs is required.

Here, we used anti-CD25-Ce6-targeted PDT, in which the anti-CD25-Ce6 complex selectively binds to tumor-infiltrated CD4^+^Foxp3^+^ Tregs and kill them locally in the tumor microenvironment by irradiation. The conjugation of Ce6 to anti-CD25 did not alter the binding activity of CD25 and effectively induced Treg apoptosis, affecting 60–70% of the Treg population 15 h after anti-CD25-Ce6-targeted PDT. Tumor-infiltrated CD4^+^Foxp3^+^ Tregs highly express CD25, while CD4^+^Foxp3^-^ cells did not, allowing the anti-CD25-Ce6 complex to specifically target tumor-infiltrated Tregs. Therefore, local irradiation can be exploited to induce spatially selective depletion of CD4^+^CD25^+^ Foxp3^+^ Tregs. Recently, a similar approach was described that used photoactivatable silica-phthalocyanine dye (IRDye 700DX)-conjugated anti-Fab fragment of anti-CD25 to selectively deplete Tregs within MC38 murine colon adenocarcinoma and LL/2 Lewis lung carcinoma [18]. Here, we used the full form of an anti-CD25 antibody that can induce antibody-dependent cellular phagocytosis of CD25^+^ cells [22]. Even though tumor-infiltrated macrophages can induce antibody-dependent cellular phagocytosis, we observed that Treg depletion was limited to the tumor site and that anti-CD25-Ce6-targeted PDT did not alter the Treg population of the draining lymph node or the systemic immune response to influenza infection. Part of the success may be attributed to the low dose of anti-CD25-Ce6 injected intratumorally, which is only 1/20 to 1/40 of the concentration used in conventional Treg-targeted immunotherapy [35]. Treg depletion by anti-CD25-Ce6-targeted PDT induced significant inhibition in B16-F10 melanoma growth, whereas administration of unconjugated anti-CD25 antibody or isotype-Ce6 did not affect tumor growth.

Our study confirmed that CD25-Ce6-targeted PDT inhibited growth of B16-F10 tumors and increased anti-tumor immunity at the tumor site. Further studies are required to determine if the adaptive immune response generated following CD25-Ce6-targeted PDT could inhibit tumors growing at other sites. Notably, the average tumor size was 128.6 ± 17 mm^3^ at treatment initiation. We think our therapy is less effective for large tumors because the laser cannot penetrate deep inside the mass. To improve therapy efficiency, we could use a laser with longer wavelength for deeper penetration into tumor tissue. In that case, Ce6 should be replaced by another molecule that can be excited at longer wavelength. Future studies will focus on optimizing the amount of anti-CD25-Ce6 injected and the administration route to minimize systemic side effects and maximize localized and specific Treg depletion using PDT.

Off-target PDT can induce tissue damage or abnormality [12]. Here, we showed that anti-CD25-Ce6 could specifically deplete Treg in tumor tissue after PDT, thus minimizing unwanted tissue damage. Considering that other cell populations in the tumor tissue were not affected by irradiation, it is very unlikely that our therapy would cause systemic side effects. Furthermore, we also assessed whether the systemic immune system was influenced by locally administered anti-CD25 antibody because systemic depletion of Treg cells could alter systemic immunity and cause severe side effects. However, as shown in Figure 5 and Supplementary Figure 2, we observed that the adaptive immune response to influenza infection was not affected after our therapy. These results show that local administration of anti-CD25-Ce6 can specifically deplete target cells without affecting other cell populations or causing systemic side effects.

Treg depletion induces tumor rejection by enhancing infiltration and activation of CD8^+^ T cells in tumors [7, 28]. Tregs reduce CD8^+^ T-cell expansion and effector differentiation, resulting in reduced production of effector cytokines and cytotoxicity through various mechanisms [36]. In particular, it is known that Treg decreases T cell polyfunctionality [37]. CD107a (lysosomal-associated membrane protein 1, LAMP1) is one of the major markers of polyfunctional T cells that is expressed on the surface of cells undergoing degranulation. CD107a also serves as a marker of cytotoxicity, and CD107a+ CD8+ T cells exhibit anti-tumor activity in an antigen-specific manner [37]. Therefore, CD8+T cell polyfunctionality is very closely associated with the anti-tumor effect *in vivo* [38].

Our findings are consistent with previous studies that used a genetically engineered Treg depletion mouse model and an antibody-targeted therapy model. These studies showed that immune-suppressive tumor microenvironments can be altered. Similarly, anti-CD25-Ce6-targeted PDT altered the immune-suppressive tumor microenvironment via Treg depletion that induced IFN-γ production in CD8^+^ T cells and increased CD8^+^ T-cell polyfunctionality, which is also in accordance with previous findings [27, 30].

Furthermore, Tregs maintain immune homeostasis by limiting T-cell responses to self, environmental, and pathogen-associated antigens and by modulating immune responsiveness [33]. Because systemic Treg depletion can induce hyper-immune responses and autoimmune responses, a local and specific Treg depletion strategy is required for cancer immunotherapy. Our anti-CD25-Ce6-targeted PDT did not affect systemic immune responses to pathogens, as shown by unaltered immune responses against influenza infection. Thus, we demonstrated that antibody-targeted PDT can be used for targeting specific cell populations within tissues without inducing severe systemic side effects.

Here, we used Ce6 conjugated to an anti-CD25 monoclonal antibody for phototherapeutic depletion of tumor-associated Tregs. However, other markers for targeting tumor-infiltrated Tregs, such as OX40 and CTLA-4, are also available [39]. Thus, further studies will examine the use of these surface molecules as targets for antibody-targeted PDT for the depletion of Tregs. Furthermore, it is important to understand the functions of immune cells other than Tregs within tumor microenvironments to develop effective immune therapies. Although our study focused on Tregs, other immune cells, such as myeloid-derived suppressor cells and tumor-associated macrophages, play immune-suppressive roles within tumor tissues [40]. Thus, future studies will apply antibody-targeted PDT to similarly study the roles of these cells.

**Figure 6:**
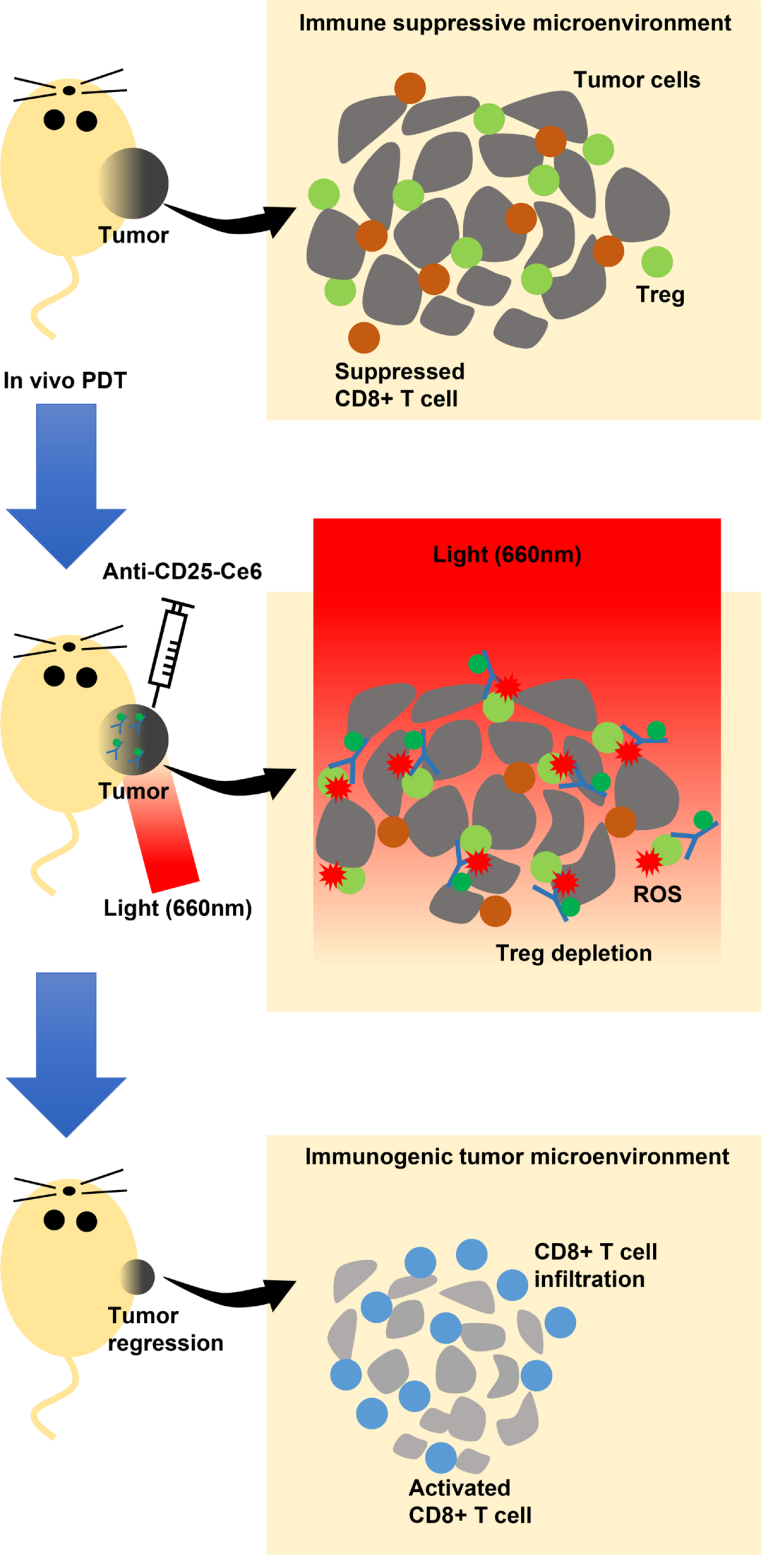
Scheme of the proposed mechanism of intratumoral Tregs-targeted photodynamic immunotherapy to induce anti-tumoral immune responses in a mouse melanoma model Tregs (green circle) suppress CD8^+^ T cell (brown circle) activation infiltrated within the tumor microenvironment, which is permissive for growth (upper panel). Mice are injected intratumorally with anti-CD25-Ce6. PDT with a 660-nm laser is applied to the injection site so that Tregs in the tumor site are specifically depleted by the ROS generated by anti-CD25-Ce6, thus modulating the local immune environment (middle panel). Activated CD8^+^ T cells (blue circle) infiltrated into the tumor microenvironment can attack tumors, inhibiting their growth (lower panel).

In conclusion, we showed that PDT targeting CD25^+^Foxp3^+^ tumor-infiltrated Tregs could achieve selective depletion of Tregs within the tumor microenvironment without inducing systemic side effects in a mouse melanoma model. After Treg depletion, CD8^+^ T-cell infiltration and activation increased within tumor tissues, and tumor growth was inhibited (Figure 6). Thus, we demonstrated that anti-CD25-Ce6-targeted PDT could specifically modulate the tumor microenvironment by depleting Tregs, making it a novel option for tumor immunotherapy.

## MATERIALS AND METHODS

### Mice

C57BL/6-Tg (Foxp3-GFP) 90Pkraj/J mice were purchased from Jackson Laboratory. Specific pathogen-free (SPF)-conditioned 8- to 12-week-old C57BL/6 male mice were purchased from DBL Co. Ltd Korea. All mice were bred in a SPF facility and animal care and housing occurred in the SPF facility of the Korea Advanced Institute of Science and Technology (KAIST). All animal procedures were in agreement with the guidelines and protocols (KA2016-19) for rodent research provided by the Institutional Animal Care and Use Committee of KAIST.

### Synthesis of the anti-CD25-Ce6 conjugate

For the conjugation of Ce6 (Santa Cruz Biotechnology) to anti-mouse CD25 antibody (Clone PC61, BioLegend) and rat IgG1λ isotype antibody (BioLegend), the carboxylic groups of Ce6 were activated by reacting Ce6 dissolved in anhydrous DMSO (5 mg/ml; Sigma-Aldrich) with equimolar N-hydroxysuccinimide (Sigma-Aldrich) and 1-ethyl-3-(3-dimethylaminopropyl) carbodiimide (Sigma-Aldrich) dissolved in DMSO (5 mg/ml) for 1 h at room temperature. The DMSO solution was then mixed with antibody solution (0.5 mg/ml) for 2 h at room temperature. The ratio between Ce6 and antibody was 100 to 1. The unbound Ce6 molecules were removed using PD-10 desalting columns (GE Healthcare). The number of Ce6 conjugated to each antibody molecule was estimated by measuring the absorbance of Ce6; approximately 10 Ce6 molecules were conjugated to each antibody molecule. The fluorescence spectra of anti-CD25 and anti-CD25-Ce6 were measured using Gemini XPS spectrofluorometer (Molecular Devices).

### Protein gel electrophoresis

To determine the anti-CD25-Ce6 conjugate molecular weights, 3 μg anti-CD25 or anti-CD25-Ce6 mixed with SDS-PAGE loading buffer (LPS solution) was boiled at 100°C for 10 min. Samples were run on SDS-PAGE (10% gel) and developed with Coomassie blue dye solution. Molecular weights of each sample were determined with Xpert Prestained protein marker (GenDEPOT).

### Anti-CD25-Ce6 conjugate binding assay

To see the binding of anti-CD25-Ce6 conjugates to CD4^+^ Foxp3^+^ cells *in vitro*, CD4^+^ T cells were enriched from Foxp3-GFP mouse spleens using a MagniSort Mouse CD4 T-cell Enrichment Kit (eBioscience). Foxp3^+^ cells were then sorted with a FACSAria II (BD Biosciences). These cells were stained with 5 μg anti-CD25-Ce6 or isotype-Ce6 on ice for 25 min. Next, these cells were washed and stained with Goat APC conjugated-anti-mouse IgG (Jackson Immuno Research). Anti-CD25-Ce6 conjugate binding was monitored with a FACSCalibur (BD Biosciences).

To measure the binding of anti-CD25-Ce6 conjugate to CD4^+^ Foxp3^+^ cells *in vivo*, B16-F10 mouse melanoma cells were subcutaneously transplanted into Foxp3-GFP mice. Anti-CD25-Ce6 or isotype-Ce6 was injected intratumorally 12 days after B16-F10 transplantation, and tumor masses were dissected 30 min after injection. Tumor masses were minced with a razor blade and digested with a mixture of 2 mg/ml Collagenase IV (Worthington Biochemical Corp) and 30 μg/ml DNase I (Roche) in PBS for 30 min at 37°C. The digested tumors were passed through 70-μm cell strainers (SPL). After cell suspensions were washed with PBS, red blood cells were depleted with ACK lysis buffer. Next, cell suspensions were washed and stained with Goat APC conjugated-anti-mouse IgG (Jackson Immuno Research). Anti-CD25-Ce6 conjugate binding was monitored with a FACSCalibur.

### *In vitro* apoptosis assay

CD4^+^ T cells were enriched from Foxp3-GFP mouse spleens using a MagniSort Mouse CD4 T-cell Enrichment Kit (eBioscience). Foxp3^+^ cells were then sorted for using a FACSAria II (BD Biosciences). Sorted cells were stained with 5 μg of anti-CD25-Ce6 or isotype-Ce6 on ice for 25 min. Stained cells were washed and 5 × 10^4^ cells were plated in each well of 96-well flat-bottom plates (FALCON) in RPMI-1640 media (Welgene) with 10% fetal bovine serum (Welgene). Plates were centrifuged for 5 min at 1500 rpm. Cells in each well were irradiated with a 660-nm laser (100 mW/cm^2^) (Micro Laser Systems) for 5 min. Cell apoptosis after 30 min or 15 h of incubation was measured by detecting surface annexin V (BioLegend) expression by a FACSCalibur (BD Biosciences).

### *In vivo* PDT

B16-F10 mouse melanoma cells were cultured in Dulbecco's Modified Eagle Medium (Welgene) containing 10% fetal bovine serum (Welgene) and 1% penicillin/streptomycin (Welgene). Once cells reached approximately 80–90% confluency, cells were harvested using Trypsin/EDTA (Welgene) and rinsed with PBS (GenDEPOT). The flanks of mice were subcutaneously injected with 5 × 10^5^ cells in 100 μl PBS. Ten days after tumor inoculation, mice were randomized into four groups that were injected with 20 μl of PBS (*n* = 6), anti-CD25-Ce6 (*n* = 6), isotype-Ce6 (*n* = 5), or free anti-CD25 (*n* = 6) intratumorally four times total at 2-day intervals. The tumors were injected with anti-CD25-Ce6 or isotype-Ce6 and irradiated (660 nm, approximately 100 mW/cm^2^, 30 min) by laser 30 min after injection. Tumor volumes were measured at 3-day intervals for 24 days by a blinded investigator with calipers using the following equation: Tumor volume = (L × W^2^)/2, where L indicates the length of the long side and W indicates that of the short side.

### Intracellular cytokine staining and flow cytometric analysis

To determine the anti-tumor immune response following PDT, *in vivo* PDT was performed twice on two separate groups of mice. Mice were sacrificed using carbon dioxide gas from the last PDT treatment. Tumor masses were isolated, minced with a razor blade, and digested with a mixture of 2 mg/ml Collagenase IV (Worthington Biochemical Corp) and 30 μg/ml DNase I (Roche) in PBS for 30 min at 37°C. The digested tumors were passed through 70-μm cell strainers (SPL). After cell suspensions were washed with PBS, red blood cells were depleted by ACK lysis buffer. To monitor CD4^+^ or CD8^+^ T-cell infiltration, cells were stained with anti-mouse CD4 (Clone GK1.5, BioLegend), anti-mouse CD8α (Clone 53-6.7, BioLegend), anti-mouse CD11b (Clone M1/70, BD Biosciences), anti-mouse CD45.2 (Clone 104, BioLegend), and anti-mouse CD3ε (Clone 145-2C11, eBioscience). Dead cells were excluded using DAPI staining. All samples were acquired on an LSR II Cell Analyzer (BD Biosciences).

Intracellular cytokine staining was performed using previously described methods [41, 42]. Briefly, cells were incubated with 50 ng/mL phorbol myristate acetate (Sigma-Aldrich), 1 μg/mL ionomycin (Sigma-Aldrich), 2 μM GolgiStop (BD Biosciences), and 2 μM GolgiPlug (BD Biosciences) for 5 h in a 37°C incubator. Cells were then stained with anti-mouse CD4, anti-mouse CD8α, anti-mouse CD11b, anti-mouse CD44 (Clone IM7, Tonbo Biosciences), and anti-mouse CD45.2, and then fixed and permeabilized using a Cytofix/Cytoperm Kit (BD Biosciences) according to the manufacturer's protocol. To detect intracellular cytokines, IFN-γ antibody (Clone XMG1.2, BD Biosciences) and CD107a antibody (Clone1D4B, BD Biosciences) were used. All samples were acquired on an LSR II Cell Analyzer and data were analyzed using FlowJo software (Tree Star).

### PR8-specific pentamer staining

To examine cytotoxic T-cell responses against lung PR8 influenza infection following *in vivo* PDT, tumor-bearing mice were intranasally infected with PR8 virus (25 PFU/mouse) 5 days after B16-F10 inoculation. Mice were treated with anti-CD25-Ce6-targeted PDT 6 days after infection with PR8. Then PDT was carried out 6 days after PR8 infection as described above. After two PDT treatments given at a 2-day interval, mice were sacrificed with carbon dioxide gas, and the lungs were isolated. Lungs were minced with a razor blade and digested with a mixture of 2 mg/ml collagenase IV and 30 μg/ml DNase I in Dulbecco's Modified Eagle Medium for 30 min at 37°C. The digested lung tissue was passed through 70-μm cell strainers. The cell suspensions were washed with PBS, and red blood cells were depleted with ACK lysis buffer. Cells were stained with anti-mouse CD8α, anti-mouse CD11b, anti-mouse CD45.2, anti-mouse CD44, and PR8 NP_366-374_ pentamer (ProImmune). All samples were acquired on an LSR Fortessa Cell Analyzer (BD Biosciences), and data were analyzed using FlowJo software.

### Serum antibody titration

To examine humoral immune responses to PR8 infection after *in vivo* anti-CD25-Ce6-targeted PDT, tumor-bearing mice were infected with PR8 (10 PFU) and subjected to anti-CD25-Ce6-targeted PDT as described above. Mice were sacrificed 14 days after PR8 infection with carbon dioxide gas. To obtain mouse serum, blood was collected via heart puncture and allowed to clot at 37°C for 1 h. The serum was prepared by centrifugation for 10 min at 10000 rpm and stored in −80°C. To determine the amount of PR8-specific IgG1 and IgM in serum, MaxiSorp 96-well plates (Nunc) were coated with formalin-fixed PR8, and diluted sera were added to detect the serum levels of PR8-specific IgG1 and IgM antibodies. The presence of PR8 antibodies was detected with horseradish peroxidase-conjugated goat anti-mouse IgG1 (Jackson Laboratory) or horseradish peroxidase-conjugated goat anti-mouse IgM (Life Technologies) antibodies. Color was developed in the plates with 3,3,5,5’-Tetramethylbenzidine (eBioscience) and stopped with H_2_SO_4_ buffer. Absorbance at 450 nm was measured with an iMark™ Microplate Absorbance Reader (Bio-Rad).

### Statistical analysis

Data are expressed as the means ± standard error of the mean (SEM). Differences among groups were analyzed using unpaired, two-tailed Student's *t* tests. Tumor growth was analyzed using two-way ANOVA tests. Statistical analysis was performed using GraphPad Prism software (GraphPad). Differences were considered statistically significant when *P* < 0.05, and are indicated as: *P < 0.05, **P < 0.01, and ***P < 0.001.

## SUPPLEMENTARY MATERIALS FIGURES



## Abbreviations

Ce6: Chlorin e6
DMSO: dimethyl sulfoxide
PDT: photodynamic therapy
ROS: reactive oxygen species
SEM: standard error of the mean
SPF: specific pathogen-free
Tregs: regulatory T cells
