# Supplementary material for: Intratumoral depletion of regulatory T cells using CD25-targeted photodynamic therapy in a mouse melanoma model induces antitumoral immune responses
Source: Oncotarget. 2017 May 7;8(29):47440–53. doi: 10.18632/oncotarget.17663 (PMC5564577; doi:10.18632/oncotarget.17663)
Supplement: Supplementary file 1 [file oncotarget-08-47440-s001.pdf]

Intratumoral depletion of regulatory T cells using CD25-targeted photodynamic therapy in a mouse melanoma model induces anti-tumoral immune responses

Supplementary Materials

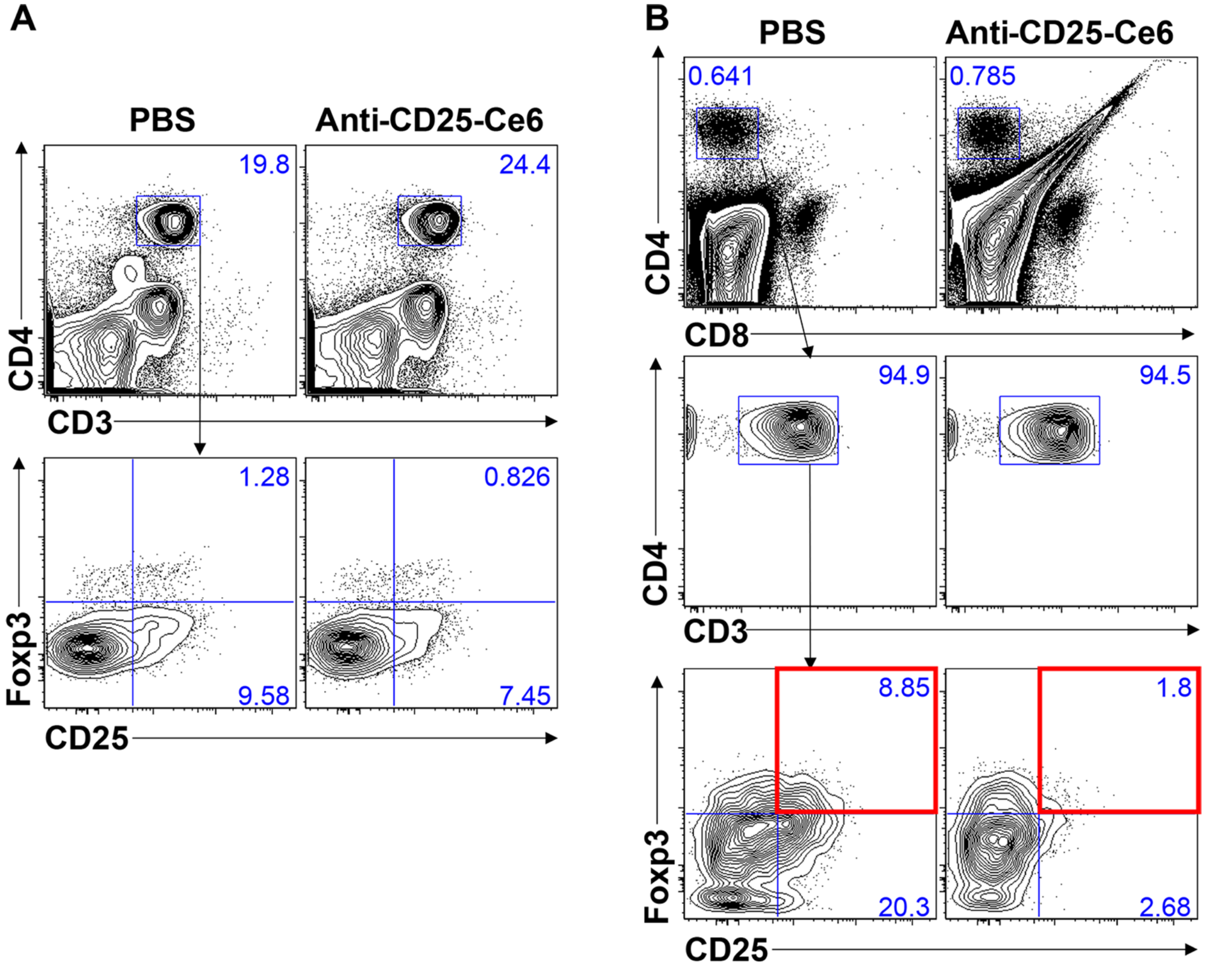

Supplementary Figure 1: Anti-CD25-Ce6-targeted PDT depletes intratumoral CD4+ CD25$^+$ Foxp3$^+$ Treg and CD4$^+$ CD25$^+$ T cells *in vivo*. Ten days after B16-F10 melanoma cell transplantation, anti-CD25-Ce6 or PBS was injected intratumorally, and tumors were irradiated with a 660-nm laser for 20 min. (**A**) Draining lymph node and (**B**) Intratumoral CD4$^+$ CD25$^+$ Foxp3$^+$ Tregs and CD4$^+$ CD25$^+$ T cells were monitored using flow cytometry.

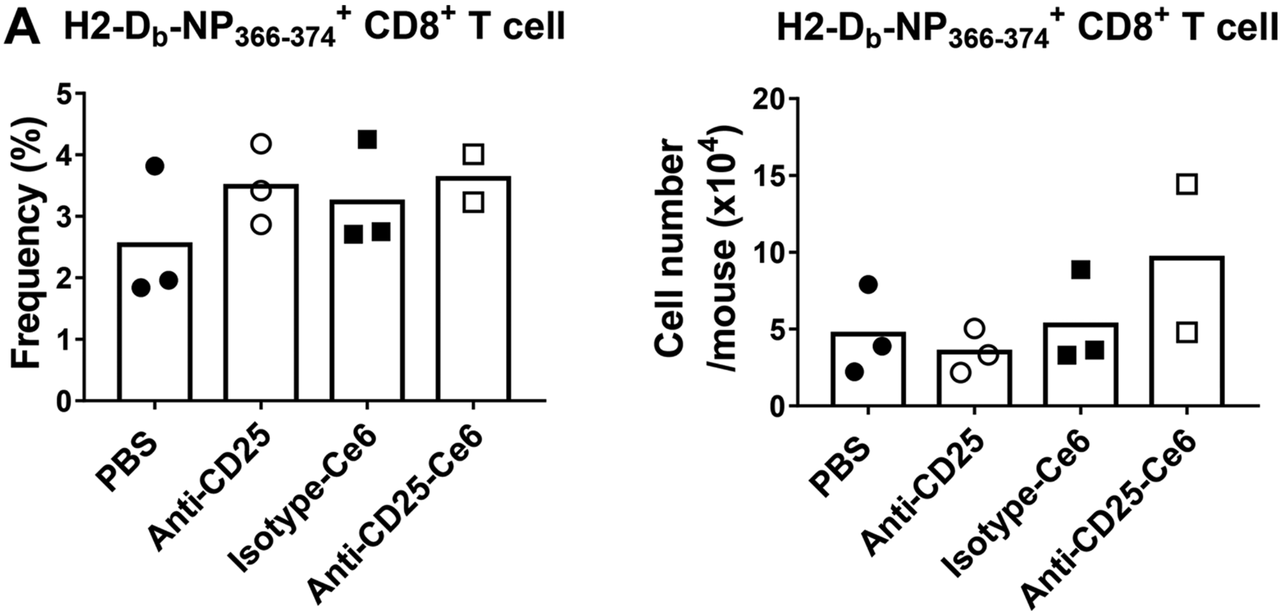

Supplementary Figure 2: Anti-CD25-Ce6-targeted PDT did not affect the PR8-specific CD8$^+$ T cell response. (**A**) B16-F10 tumor-bearing mice were injected intratumorally with PBS, anti-CD25 antibody, isotype-Ce6, or anti-CD25-Ce6, and PDT was performed twice on a 2-day interval. Next, 25 PFU of PR8 was intranasally administered after PDT completion. PR8 NP$_{366\text{-}374}$-specific CD8$^+$ T cells in lungs were monitored using flow cytometry with the H2-Db-NP$_{366\text{-}374}$ pentamer 7 days after PR8 infection. Data are shown as bar graphs of the frequency of PR8 NP366-374-specific CD8$^+$ T cells; each dot represents an individual mouse.
